# Genetic diversity and relationship between domesticated rye and its wild relatives as revealed through genotyping‐by‐sequencing

**DOI:** 10.1111/eva.12624

**Published:** 2018-03-26

**Authors:** Mona Schreiber, Axel Himmelbach, Andreas Börner, Martin Mascher

**Affiliations:** ^1^ Leibniz Institute of Plant Genetics and Crop Plant Research (IPK) Gatersleben Seeland Germany; ^2^ German Centre for Integrative Biodiversity Research (iDiv) Halle‐Jena‐Leipzig Leipzig Germany

**Keywords:** crop wild relatives, crop‐wild hybridization, gene flow, genebank collections, genotyping‐by‐sequencing, population genomics, rye, *Secale cereale*

## Abstract

Rye (*Secale cereale* L.) is a cereal grass that is an important food crop in Central and Eastern Europe. In contrast to its close relatives wheat and barley, it was not a founder crop of Neolithic agriculture, but is considered a secondary domesticate that may have become a crop plant only after a transitory phase as a weed. As a minor crop of only local importance, genomic resources in rye are underdeveloped, and few population genetic studies using genomewide markers have been published to date. We collected genotyping‐by‐sequencing data for 603 individuals from 101 genebank accessions of domesticated rye and its wild progenitor *S. cereale* subsp. *vavilovii* and related species in the genus *Secale*. Variant detection in the context of a recently published draft sequence assembly of cultivated rye yielded 55,744 single nucleotide polymorphisms with present genotype calls in 90% of samples. Analysis of population structure recapitulated the taxonomy of the genus *Secale*. We found only weak genetic differentiation between wild and domesticated rye with likely gene flow between the two groups. Moreover, incomplete lineage sorting was frequent between *Secale* species because of either ongoing gene flow or recent speciation. Our study highlights the necessity of gauging the representativeness of ex situ germplasm collections for domestication studies and motivates a more in‐depth analysis of the interplay between sequence divergence and reproductive isolation in the genus *Secale*.

## 1 INTRODUCTION

Rye (*Secale cereale* L.) is, after wheat, the second most commonly used cereal for bread making in Europe. It is a diploid (2*n* = 14) grass of the Triticeae tribe, which also includes wheat (*Triticum* spp.) and barley (*Hordeum vulgare* L.). The major rye‐growing regions of the world are Central and Eastern Europe (http://www.fao.org/faostat), whose colder climate favors the high frost tolerance of rye and its ability to thrive on poor soils. Under such adverse growth conditions, rye is higher yielding than wheat. The closely related cereal crops wheat, barley, and rye share many characteristics: (i) The center of diversity of the wild progenitors of these three crops is the Fertile Crescent and neighboring regions (Zohary, Hopf, & Weiss, 2012); and (ii) all Triticeae have a haploid set of seven chromosomes and very repeat‐rich genomes that are highly collinear to each other (Bauer et al., 2017; Devos, Millan, & Gale, 1993). Unlike wheat and barley, rye is not considered a founder crop of Neolithic agriculture (Abbo, Lev‐Yadun, Heun, & Gopher, 2013). Although archaeobotanical remains of rye were found in early Neolithic sites in the Northern Levant, their domestication status is uncertain (Hillman, 1978; Nesbitt, 2002). Substantial numbers of rye grains appear in the archaeological record only in the European Bronze Age (Hartyanyi & Novaki, 1975). By the Iron Age, rye has likely become a widely used crop (Behre, 1992). A commonly evoked explanation for this delayed rise to agricultural relevance is that rye spread as a “hitchhiking” weed in wheat and barley fields (Zohary et al., 2012) after adaptation to an agricultural habitat. According to this hypothesis of Vavilovian mimicry (McElroy, 2014), rye domestication may have proceeded in two stages: First, wild rye became a weed that may have already had acquired key domestication traits such a tough rachis and larger seeds to facilitate co‐harvesting with wheat and barley. Later, when its superior performance under inclement Northern climate became evident, farmers started to sow and harvest rye in its own right, turning the preadapted weed into a “fully domesticated” crop. Because of this complex history, rye is considered a secondary domesticate (Preece et al., 2017).

Rye belongs to the small genus *Secale* with only three taxa*,* which contains the putative wild progenitor, *S. cereale* subsp. *vavilovii* together with domesticated rye *S. cereale* subsp. *cereale* as well as several other described subspecies (possibly weedy), and two other wild species *S. strictum* and *S. sylvestre* (Frederiksen & Petersen, 1998). Among these, only rye is used as a crop in current times, although *S. strictum* may have been used as a forage crop (Hammer, Skolimowska, & Knüpffer, 1987). The species in the genus *Secale* differ in life cycle and breeding system (Hammer, 1990). Rye is an annual outcrosser, but self‐compatibility has been reported both in its wild progenitor *S. cereale* subsp. *vavilovii* (Hammer et al., 1987) and in breeding lines of cultivated rye (Voylokov, Fuong, & Smirnov, 1993). *S. strictum* and *S. sylvestre* are perennial outcrossers and annual selfers, respectively (Frederiksen & Petersen, 1998). The literature abounds with proposed infraspecific taxa for *S. cereale* and *S. strictum* that reflect differences in geographic range, growth habit (e.g., weediness), and morphological characters such as hairiness of leaf sheath of spike brittleness (Frederiksen & Petersen, 1998). Artificial interspecific hybridizations are possible between all rye taxa, although crossability of *S. sylvestre* with other species is low (Khush, 1962; Khush & Stebbins, 1961). Low fertility of the hybrids is common and is possibly caused by chromosomal translocations (Singh & Röbbelen, 1977; Stutz, 1957). Spontaneous hybridizations between cultivated *S. cereale* and neighboring wild‐growing *S. strictum* populations have been reported for Italian and Anatolian sites (Perrino, Hammer, & Hanelt, 1984; Zohary, 1960).

The agronomical importance of rye as a cereal crop is eclipsed by the prominent role of its close relatives wheat and barley in modern agriculture. Reasons for this include easier line breeding in the self‐fertilizing crops wheat and barley and a preference for wheat for baking and barley for malting in most regions of the world. Thus, comparatively few resources have been allocated to set up a genomic infrastructure comprising reference genome, representative diversity panels, and high‐throughput marker technologies. Recently, Bauer et al. (2017) published an annotated draft reference genome sequence assembly constructed for the inbred line Lo7. This assembly represents only 2.8 Gb of the 8‐Gb genome and is fragmented into 1.3 million sequence scaffolds of which only 158 Mb were anchored to chromosomal positions. Despite these shortcomings, this draft assembly will likely serve as an important reference anchor for population genomic studies in the same way as the genetically anchored sequence assemblies of barley and wheat (International Barley Genome Sequencing Consortium, 2012; International Wheat Genome Sequencing Consortium, 2014) underpinned genomewide surveys of sequence diversity (Jordan et al., 2015; Russell et al., 2016). The progress in genomics technology has enabled the development of high‐throughput genotyping platforms also for minor crops. Mining transcriptome sequence data from five winter rye breeding lines, Haseneyer et al. (2011) designed a SNP array with 5,234 features. A subset of these were used to genotype a diversity panel of rye and wild relatives by Hagenblad, Oliveira, Forsberg, and Leino (2016). Compared to SNP arrays, reduced‐representation sequencing methods based on digestion with restriction enzymes have the advantage of joint discovery and genotyping of sequence variant without the requirement for ascertaining polymorphic markers in a discovery panel, which may lead to underestimation of genetic diversity of diverse genetic material. A flavor of reduced‐representation sequencing, DArTseq (Li et al., 2015), has been previously employed for linkage mapping in rye (Milczarski, Hanek, Tyrka, & Stojałowski, 2016; Rakoczy‐Trojanowska et al., 2017).

Here, we report the analysis of single nucleotide polymorphism (SNP) data of rye and its wild relatives obtained through genotyping‐by‐sequencing (GBS; Elshire et al., 2011). We performed explorative population genetic analysis of this dataset. Our results support the *Secale* taxonomy of Frederiksen and Petersen (1998) with little infraspecific substructure in *S. cereale* and recently shared ancestry between taxa.

## 2 MATERIALS AND METHODS

### 2.1 Plant material

One hundred and one genebank accessions from the *Secale* taxa (domesticated rye [*S. cereale* subsp. *cereale*], *S. cereale* subsp. *vavilovii*, further *S. cereale* subspecies, *S. strictum*, and *S. sylvestre)* were selected based on passport information (taxonomic status, country of origin, collection site) available in the genebank information system of the German Federal ex situ Genebank at IPK Gatersleben (GBIS; https://gbis.ipk-gatersleben.de/GBIS_I, Oppermann, Weise, Dittmann, & Knupffer, 2015). Seeds from the selected accessions were obtained from the IPK genebank. Passport information from IPK's genebank information system is given in Table S1, and collection sites are shown in Figure S1. DNA was extracted from leaf tissue of six plants at the seedling stage using the DNeasy Plant Mini Kit (Qiagen, Hilden, Germany). Three plants of each accession were grown to full maturity to observe seed shattering.

### 2.2 Genotyping‐by‐sequencing

Genotyping‐by‐sequencing libraries were prepared for the six individually bar‐coded plants of each accession using the PstI‐MspI two‐enzyme approach (Poland, Brown, Sorrells, & Jannink, 2012) as described previously (Wendler et al., 2014). Libraries were sequenced (single read, 100 cycles) on the Illumina HiSeq 2500 device at IPK Gatersleben (Wendler et al., 2014). Raw data have been deposited in the European Nucleotide Archive under accession number PRJEB22681. Accession numbers for individual samples are provided in Table S2.

### 2.3 Read mapping and variant calling

Primary data analysis followed the procedures of Mascher, Wu, Amand, Stein, and Poland (2013). Briefly, after adapter trimming with Cutadapt (Martin, 2011), reads were mapped to the whole‐genome shotgun sequence assembly of rye cultivar Lo7 (Bauer et al., 2017) using BWA‐MEM version 0.7.13 (Li, 2013). The alignments were converted to BAM format with SAMtools (Li et al., 2009) and sorted with NovoSort (http://www.novocraft.com/products/novosort/). Variant calling was performed with SAMtools and BCFtools version 1.3 (Li, 2011) using a mapping quality threshold of 30 and a base quality threshold of 20. The resultant VCF file was filtered with a previously published AWK script (Mascher et al., 2013). We considered only biallelic sites. Sites with a quality score below 40 were discarded. Genotype calls (inferred allelic states for individuals at segregating sites) as reported by BCFtools were set to missing if either the read depth (DP) or the genotype quality (GQ) was below 5. Subsequently, sites with more than 90% missing data or more than 90% heterozygous calls were discarded. The resulting marker‐by‐individual matrix was imported into the R statistical environment (R Core Team, 2015) and further analyzed using functionalities of R packages data.table (https://cran.r-project.org/web/packages/data.table/), SNPRelate (Zheng et al., 2012), and SeqArray (Zheng et al., 2017). We further filtered the SNP set to include only sites with up to 10% missing across all samples. Three samples (belonging to the accessions R1112, R193, and R918) with less than 80% present data in the final call set were excluded from further analysis. Read depth analyses were performed using the command “samtools depth” (Li et al., 2009). The SNP matrix was deposited at the Plant Genomics and Phenomics Research Data Repository (Arend et al., 2016) under Digital Object Identifier (https://doi.org/10.5447/ipk/2018/1). DOIs were registered with e!DAL (Arend et al., 2014).

### 2.4 Population genetic analyses

Principal component analysis was performed with EIGENSOFT version 6.0.1 (Patterson, Price, & Reich, 2006) using least‐square projection and disabling outlier removal. Fixation indices (F_ST_) were calculated on previously defined groups using the modified Hudson estimator (Hudson, Slatkin, & Maddison, 1992) using the formulas of Bhatia, Patterson, Sankararaman, and Price (2013). Neighbor‐joining trees were constructed with the R package “ape” (Paradis, Claude, & Strimmer, 2004). Pairwise identities based on identity‐by‐state were computed using SNPRelate (Zheng et al., 2012). Model‐based ancestry estimation was performed with ADMIXTURE (Alexander, Novembre, & Lange, 2009). For each analysis, twenty replicate runs per cluster size K were performed and combined with CLUMPP (Jakobsson & Rosenberg, 2007). D‐statistics were calculated with AdmixTools (Patterson et al., 2012) using wheat as the outgroup. To determine ancestral states, SNP positions were lifted to wheat. Toward this purpose, 300‐bp regions flanking each SNP were extracted from the genome sequence assembly of rye (Bauer et al., 2017) and aligned to the genome sequence assembly of bread wheat (International Wheat Genome Sequencing Consortium, 2014) with BWA‐MEM (Li, 2013). Prior to mapping, the wheat assembly was divided into subgenomes based on the POPSEQ genetic map (Chapman et al., 2015) and exact SNP coordinates in wheat were determined by parsing the BAM CIGAR string (Li et al., 2009) using BEDTools (Quinlan & Hall, 2010) and R. If a flanking region was aligned to more than one wheat subgenome, only one hit was considered in the order (A, B, D). Predicted protein and transcript sequences of rye genes were aligned to their wheat counterparts (separated into subgenomes) using BLAST+ (Camacho et al., 2009). We considered only SNP positions that (i) overlapped an annotated rye gene and for which (ii) also their lifted positions in wheat overlapped the corresponding best hit in the wheat gene set. Nucleotides at these positions were extracted from the wheat assembly and used as ancestral states. To test the relationship between genetic and geographic distances, we performed a Mantel test using the R package adegenet (Jombart & Ahmed, 2011).

## 3 RESULTS

### 3.1 Genotyping‐by‐sequencing in Secale species

We obtained genotyping‐by‐sequencing data for 101 genebank accessions of domesticated rye and wild *Secale* forms. Our panel includes 81 rye accessions, five accessions of *S. cereale* subsp. *vavilovii*, eleven accessions of *S. strictum*, and four accessions of *S. sylvestre* (Table S1, Figure S1). Six individuals were sampled from each accession for multiplexed genotyping‐by‐sequencing following the protocol of Wendler et al. (2014) using bar coding of individual DNA samples. Read mapping and variant calling were performed against the draft genome sequence assembly of cultivated rye (Bauer et al., 2017) using a reference‐based SNP calling pipeline previously applied in wheat, barley, and wild relatives (Chapman et al., 2015; Mascher et al., 2013; Wendler et al., 2015). Stringent filtering for a low missing rate (10%) across all samples resulted in a set of biallelic 55,744 SNPs distributed across a target region of 2.4 Mb, that is, genomic intervals that are covered with at least five reads in 90% of the samples (Table 1). A total of 603 samples had more than 80% present data and were used for further analysis. Only 22% of SNPs were assigned to approximate chromosomal locations in the partially ordered sequence assembly (Table 1). As expected from wheat and barley (Poland et al., 2012), our choice of restriction enzymes for GBS enriched the libraries for fragments from genic regions: 29% of our SNPs were located in genic regions, although only 12% of the total assembly were annotated as genic sequence.

**Table 1:** Summary of variant calling results

	Number of SNPs	Contig length (Mb)	Target length (Mb)
All SNPs	55,744 (100%)	270	2.4
SNPs anchored to chromosomes^a^	11,294 (22%)	83	0.8
SNPs in annotated genes	14,492 (29%)	133	0.7
Anchored SNPs in genes	7,160 (14%)	57	0.3

a The genetic map of Bauer et al. (2017) was used.

### 3.2 Clear differentiation between species despite incomplete lineage sorting

**Figure 1:**
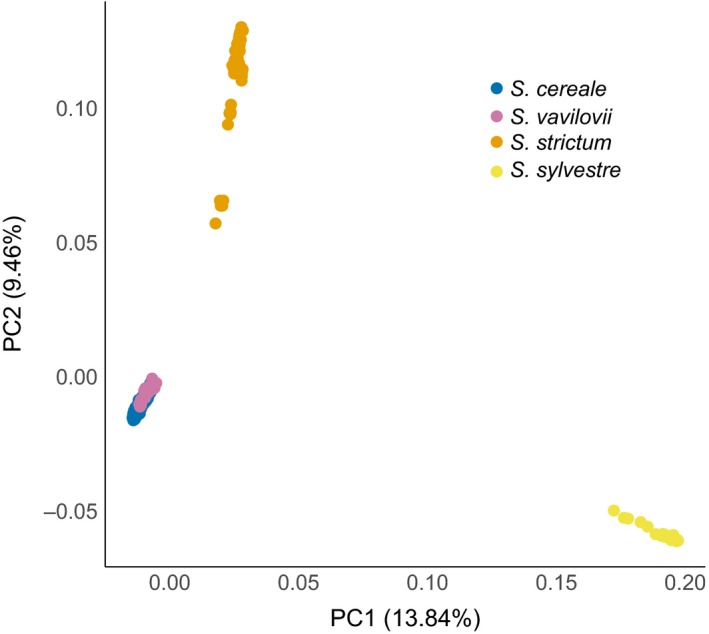
Principal component analysis across all accessions. The first two principal components (PCs) are plotted against each other. Dots correspond to individual samples, which are colored according to taxonomy. The proportion of variance explained by each PC is indicated in parentheses in the axis labels

**Figure 2:**
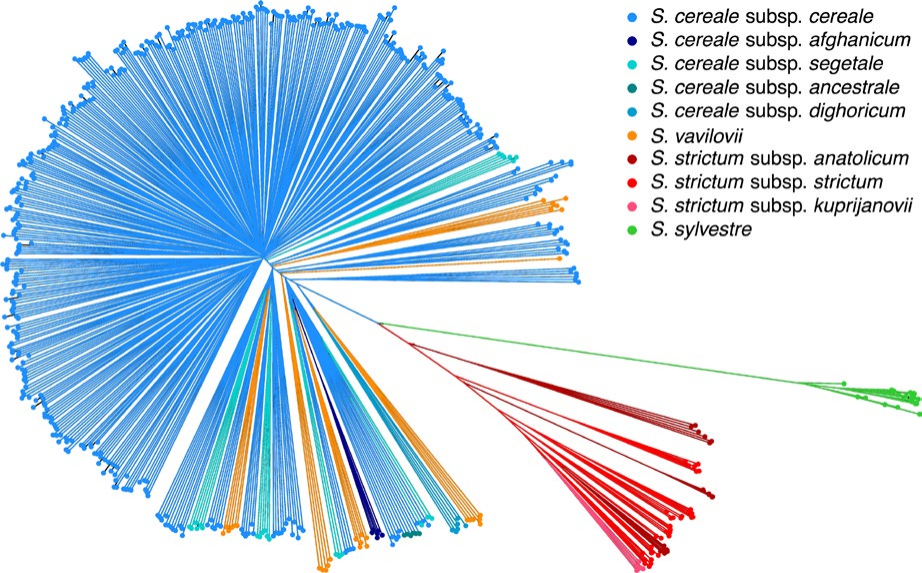
Neighbor‐joining tree. Tips and branches are colored according to taxonomy as indicated in the legend. The wild progenitor of domesticated rye *S. cereale* subsp. *vavilovii* is here indicated as *S. vavilovi*

**Figure 3:**
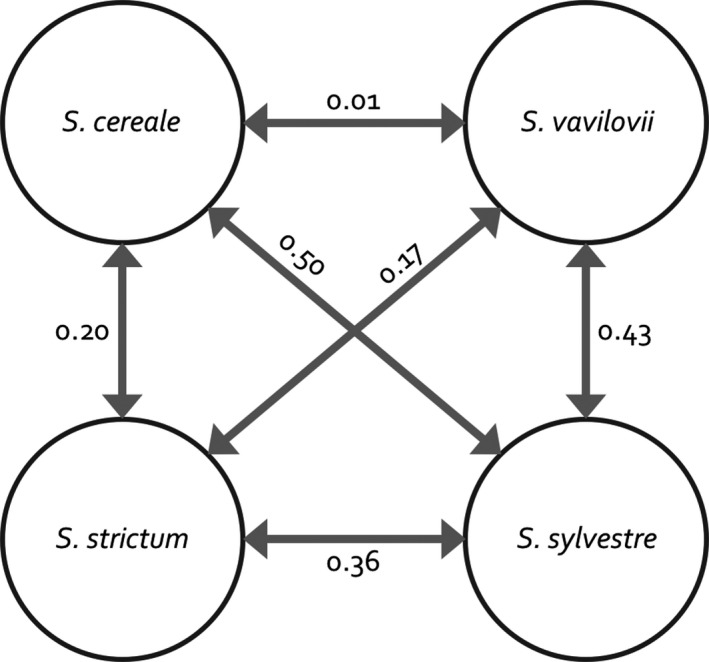
Fixation indices (F_ST_) between *Secale* taxa. F_ST_ was computed using the method of Bhatia et al. (2013). The wild progenitor of domesticated rye *S. cereale* subsp. *vavilovii* is here indicated as *S. vavilovii* for simplicity

We performed several explorative analyses, which all supported a clear differentiation of the three *Secale* species from each other. A principal component analysis (PCA; Patterson et al., 2006) across all samples separated the three species, *S. cereale*,* S. cereale* subsp. *vavilovii*, and *S. sylvestre*, in the first two principal components, explaining together 23.3% of the total variation (Figure 1). *S. cereale* subsp. *vavilovii* clustered closely together with the other *S. cereale* subspecies in the PCA plot. This pattern was recapitulated by a neighbor‐joining (NJ) tree (Figure 2), which did not separate rye and *S. cereale* subsp. *vavilovii* samples. Visual inspection of the NJ tree revealed the six samples form the tips of a common branch, indicating a high relatedness of individuals from the same accession and the genetic integrity of the analyzed ex situ accessions. This was confirmed by a relatedness analysis based on identity‐by‐state: The five closest relatives of a given sample were from the same accession as that sample, confirming the close genetic affinity of samples from the same genebank accession. Fixation indices (F_ST_) corroborated the clear separation between *Secale* species and the weak differentiation between *S. cereale* subsp. *vavilovii* and the other *S. cereale* subtaxa (Figure 3). In addition, F_ST_ values indicate a closer affinity of *S. cereal*e to *S. strictum* than to *S. sylvestre* (Figure 3).

Speciation is followed by lineage sorting, that is, the fixation of different alleles of ancestrally segregating variants in the descendant species by stochastic processes. Recent speciation events or ongoing gene flow due to incomplete fertility barriers leads to an elevated proportion of shared segregating sites between closely related species. We found a substantial number of biallelic SNPs variants for which both alleles were present in more than one species (Table 2). For example, 2,760 sites were polymorphic in both *S. cereale* and *S. sylvestre*. We note that *S. strictum* had relatively more polymorphic sites in common with *S. cereale* (65.8%) than had *S. sylvestre* (51.1%), indicating that *S. sylvestre* split first from the lineage leading to *S. strictum* and *S. cereale* in agreement with AFLP phylogeny of Chikmawati, Skovmand, and Gustafson (2005) and the chloroplast phylogeny of Petersen and Doebley (1993).

**Table 2:** Segregating sites shared between subspecies

Taxon	Polymorphic sites^a^	Polymorphic sites shared with domesticated rye^b^
*S. cereale* without subsp. *vavilovii*	36,213	
*S. cereale* subspecies *vavilovii*	26,457	25,185 (95.2%)
*S. strictum*	25,591	16,841 (65.8%)
*S. sylvestre*	5,397	2,760 (51.1%)

a Segregating sites are a subset of the universe described in Table 1 **.**

b *Secale cereale* without subspecies *vavilovii*.

### 3.3 Evidence for crop‐wild gene flow in *S. cereale*

**Figure 4:**
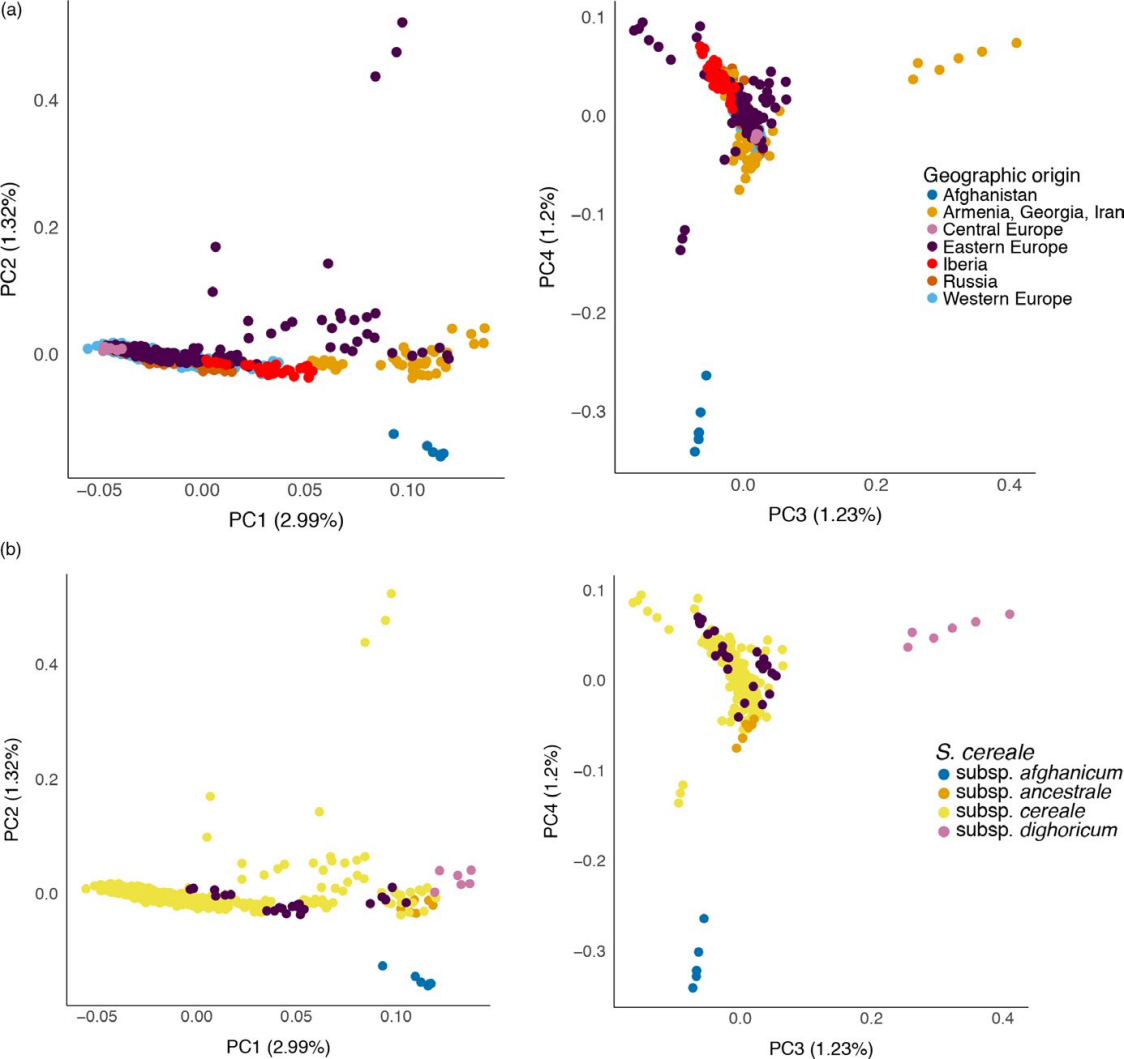
Principal component analysis across *S. cereale* samples, excluding subspecies *vavilovii*. The top (a) and bottom (b) panels show the same data using different color and symbol (as indicated in the legends) to show geographic and taxonomic patterns. The left panels plot principal component 1 (PC1) against PC2; the rights panels show PC3 versus PC4. The proportion of variance explained by each PC is indicated in parentheses in the axis labels

Studies in wild and domesticated barley have shown (i) a clear separation of the crop and its wild progenitor and (ii) a good correlation between geographic and genetic distance in the crop and, independently, the wild relative (Russell et al., 2016). To investigate the relationship between the geographic origin of our rye accessions and their relatedness, we first performed a principal component analysis (PCA) using only *S. cereale* samples without subspecies *vavilovii* (Figure 4). Samples from Western Asia are separated from European samples in the first principal component (PC) (Figure 4a). No further structure according to geography was evident in the first four PCs. Apart from a clustering of individuals from the same accession, also taxonomic classifications were not reflected in the PCA **(**Figure 4b**).** The first four components together explained only 6.7% of the variance, indicating low genetic differentiation between populations. A Mantel test on genetic distances based on pairwise identity‐by‐state analysis and the geographic origin given in the passport data revealed a weak (*r* = .09), albeit significant (*p* = .01), correlation.

**Figure 5:**
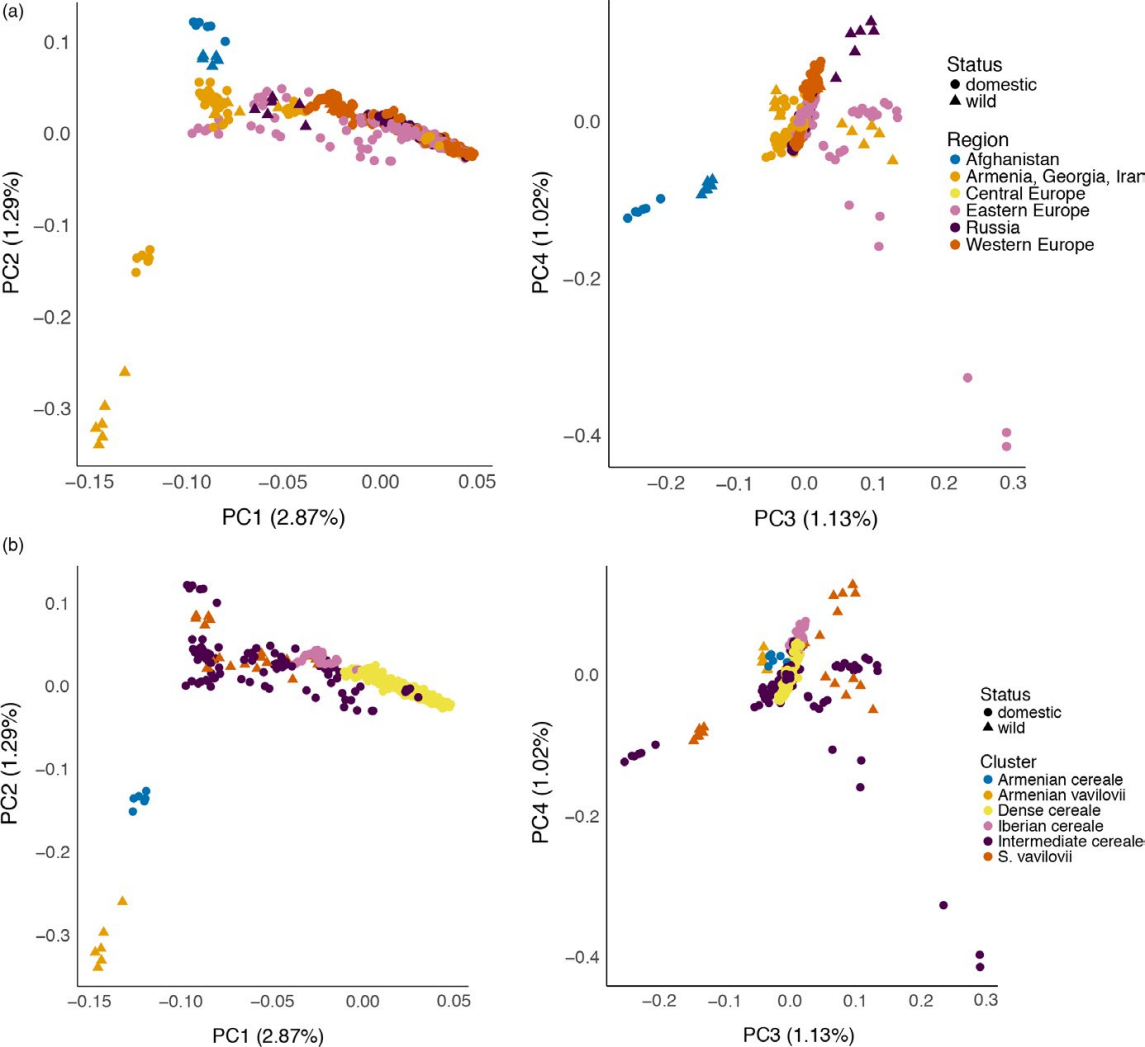
Principal component analysis across *S. cereale* samples, including subspecies *vavilovii*. The top (a) and bottom (b) panels show the same data using different color and symbol (as indicated in the legends) to indicate domestication status, geographic origin, and a custom group used to inspect patterns of allele sharing (Table 3). The left panels plot principal component 1 (PC1) against PC2; the right panels show PC3 versus PC4. The proportion of variance explained by each PC is indicated in parentheses in the axis labels

Next, we included the *S. cereale* subsp. *vavilovii* samples in the PCA (Figure 5a). Most conspicuously, samples of one accession (R1003 from Armenia) were clustered apart all the other *S. cereale* samples. One accession of domesticated rye (R2863 also from Armenia) was intermediate between R1003 and the remaining samples. We used D‐statistics (Patterson et al., 2012; Reich, Thangaraj, Patterson, Price, & Singh, 2009) to inspect allele sharing patterns of these two accessions and the other samples. We note that due to incomplete lineage sorting, both *S. strictum* and *S. sylvestre* were inappropriate outgroups, and we had to use wheat to determine ancestral states for 1,372 SNPs. We found that R1003 shared significantly (*Z* = 4.6; Table 3) more derived alleles with R2863 than with other *S. cereale* accessions. The most parsimonious explanation for this observation is gene flow between sympatric wild and domestic rye in Armenia. Our panel also includes one accession of *S. strictum* (R2859) from Armenia that also shared significantly more derived alleles with both R1003 and R2859 than with other *S. cereale* accessions (Table 3). It is tempting to speculate that gene flow between *S. strictum* and *S. cereale* subsp. *vavilovii* is still common enough to give rise to shared covariation between genetic relatedness and geography in both species.

**Table 3:** D‐statistics

P1	P2	P3	BABA^a^	ABBA^a^	D	SE^b^	Z^b^
R2863	*S. cereale* without R2863	R1003	72	47	0.21	0.05	4.58
R1003	*S. cereale* without R2863 and R1003	R2859	68	49	0.16	0.04	3.69
R2863	*S. cereale* without R2863 and R1003	R2859	65	50	0.13	0.06	2.09
“Intermediate”^c^	“Dense”^c^	*S. cereale subsp. vavilovii*	66	47	0.16	0.02	8.49
Iberia^c^	“Dense”	*S. cereale subsp. vavilovii*	61	47	0.13	0.02	7.03
Iberia	“Dense”	Armenia	62	46	0.14	0.02	6.04
Iberia	“Dense”	“Intermediate”	61	48	0.12	0.02	5.78

a Number of ABBA and BABA sites. Wheat was used as an outgroup for all comparisons.

b *SE*: standard error; Z: Z‐score.

c The composition of the “dense,” “intermediate,” Iberia, and Armenia groups of *S. cereale* is indicated in Figure 5b.

The joint PCA of wild and domesticated *S. cereale* did not split cultivated rye into clear clusters. However, we observed that wild *S. cereale* subsp. *vavilovii* samples were mixed with domesticated samples in intermediate PC1 ranges, while *S. cereale* subsp. *vavilovii* was absent from higher PC1 ranges, where a dense cluster of cultivated samples was centered. D‐statistics indicate that *S. cereale* subsp. *vavilovii* (Figure 5b) shared significantly more derived alleles (*Z* = 8.5; Table 3) with “intermediate” than “dense” accessions. Similarly, D‐statistics indicated a higher genetic affinity of *S. cereale* subsp. *vavilovii*, “intermediate” and Armenian accessions with Iberian material than with “dense” accessions (Table 3). A differentiation of Iberian and Eastern European germplasm was described before by Parat et al. (2016) based on 32 microsatellite markers and attributed to different end uses: either forage (Southern Europe) or human consumption (Eastern Europe). “Intermediate” accessions originate from the Middle East, but also from East Europe, where *S. cereale* subsp. *vavilovii* does not occur (Frederiksen & Petersen, 1998), making it difficult to decide whether allele sharing is due to recent gene flow or ancestral population structure, or both.

### 3.4 Shared ancestry components between rye and related species

**Figure 6:**
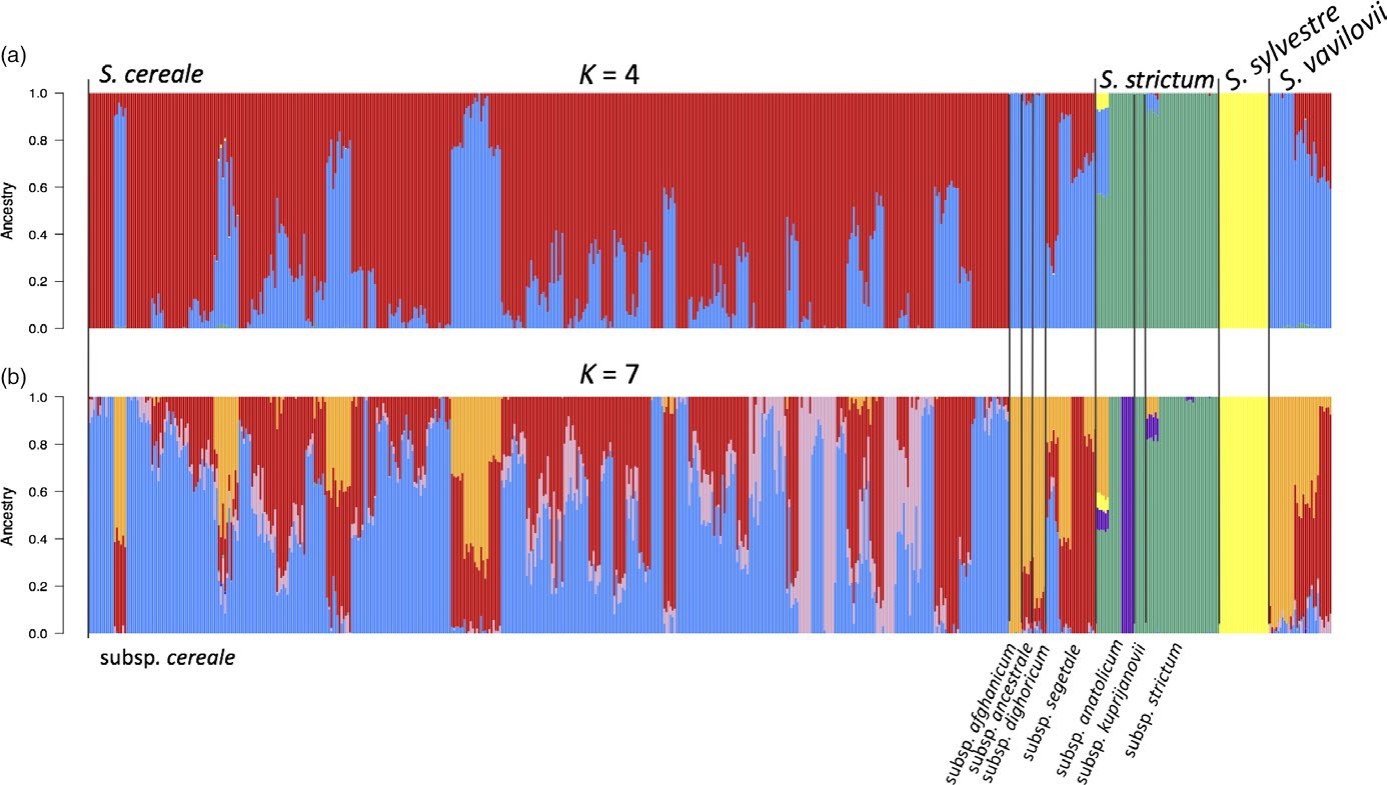
ADMIXTURE results assuming four (a) and seven (b) ancestral populations. Colors represent ancestry components. Stacked bars represent samples. Samples are arranged according to taxonomy as indicated in the *x*‐axis labels

We performed model‐based ancestry estimation across all our samples with the assumptions of either four or seven ancestral populations using ADMIXTURE (Alexander et al., 2009). Four ancestral populations capture the intuitive notion of three species and *S. cereale* divided into domesticated rye and wild *S. cereale* subsp. *vavilovii*; seven ancestral populations were suggested by the cross‐validation criterion of ADMIXTURE (Alexander et al., 2009; Figure S2). In both scenarios, ancestry components were shared between the three taxa (Figure 6). The major ancestry components of *S. cereale* subsp. *vavilovii* were also present in domesticated rye (Figure 6). When seven ancestral populations were used, two components (blue and green color in Figure 6B) were private to *S. strictum*. Interestingly, the “green” ancestry component was present in unadmixed state in a group of six individuals belonging *S. strictum* subsp. *kuprijanovii*, a subspecies from the Caucasus (Hammer et al., 1987). Additionally, this component occurred in two other *S. strictum* accessions also sharing ancestry with *S. cereale* (R2859 from Armenia and R2431 from Bulgaria). This shared ancestry could be caused by recent interspecific gene flow or shared ancestral variation. An improved, physically ordered reference genome sequence would enable the inspection of haplotype lengths to date hybridization events (Harris & Nielsen, 2013).

## 4 DISCUSSION

We have used genotyping‐by‐sequencing to discover genomewide SNP markers in a diversity panel of rye and its wild relatives. Compared to previous studies based on microsatellite markers (Parat et al., 2016), the number of genetic markers in our study is orders of magnitude higher, while at the same time allowing the rapid analysis of many samples. Hagenblad et al. (2016) used a SNP array with 576 features to genotype a panel of *S. cereale*,* S. cereale* subsp. *vavilovii*, and *S. strictum* genebank accessions and found that they likely underestimated diversity in *S. strictum* because the discovery panel comprised only domesticated rye. Genotyping‐by‐sequencing does not introduce ascertainment bias during complexity reduction, but mapping reads to a single reference genome can give rise to similar biases (Arnold, Corbett‐Detig, Hartl, & Bomblies, 2013): Any restriction fragments missing from the reference sequence assembly but present in other taxa are ignored. Vice versa, mutations in restriction sites present in the reference genome can result in missing data when more diverse germplasm is assayed. By focusing only on variant sites that have present genotype calls in at least 90% of samples, we reduce the issue of allelic dropout, while possibly underestimating genetic diversity by focusing on conserved restriction fragments (Gautier et al., 2013). However, we believe that our conclusions from explorative analyses of population structure and genetic differentiation are robust to these potential biases.

In the past, several taxa included in our study such as *S. cereale* subsp. *vavilovii* or *S. strictum* subsp. *kuprijanovii* were considered as distinct species (Roshevitz, 1947). This overclassification may trace back to a preference of crop geneticists for obvious differences in conspicuous characters that nevertheless do not warrant elevation to the species level (Harlan & de Wet, 1971). The taxonomic revision of Frederiksen and Petersen (1998) divides the genus *Secale* into three species: *S. cereale*,* S. strictum*, and *S. sylvestre* with *S. cereale* subsp. *vavilovii* as a subspecies of *S*. *cereale*. Our data confirm this grouping: The three species form well‐defined clusters in the principal component analysis, sit on distinct branches in a neighbor‐joining tree, and are assigned predominantly to different ancestry components in an ADMIXTURE analysis. The correspondence between infraspecific taxonomy and marker‐based clustering is less clear. Similar results were obtained by Hagenblad et al. (2016) who found limited genetic clustering according to taxonomy below the species level. All our samples have been maintained in ex situ genebank collections for at least one decade, some even since the 1940s. One possible explanation for the weak differentiation between *S. cereale* subtaxa is recent “artificial” admixture during genebank propagation, when domesticated and wild accessions are grown in close proximity. Although genebank managers do try to prevent cross‐pollination, previous reports have found misassigned accessions and questioned the suitability of genebank material of crop wild relatives for population genetic studies (Jakob et al., 2014). Our data, however, support the genetic integrity of the analyzed accessions: Individuals of all accessions cluster closely together in PCA plots and a neighbor‐joining tree, a pattern that would be difficult to reconcile with random outcrossing events between unrelated materials. Moreover, all individuals of uncultivated taxa (*S. vavilovii*,* S. strictum, S. sylvestre)* had shattering ears, supporting their wild‐growing status.

Interspecific gene flow in the genus *Secale* is not unexpected: Previous studies have examined the crossability of different *Secale* species and have identified chromosomal rearrangements as likely causes for frequent hybrid sterility (Hrishi & Müntzing, 1960; Singh, 1977; Stutz, 1957). However, these cytogenetic studies examined offspring of artificial interspecific crosses in a laboratory setting. Our results regarding the close affinity of Armenian samples from domesticated (*S. cereale* subsp. *cereale*) and wild rye (*S. cereale* subsp. *vavilovii*) (Figure 5) provide molecular evidence for hybridization between different taxa occurring in nature, supporting anecdotal field observations of collectors of plant genetic resources (Perrino et al., 1984). In principle, a wider wild genepool is accessible to prebreeding efforts in rye than in its close relative barley, also a diploid Triticeae. Fertile progeny is not readily obtained for interspecific crosses in *Hordeum*: Even crosses between barley and its closest relatives *H. bulbosum* frequently result in uniparental genome elimination (Houben, Sanei, & Pickering, 2011; Kasha & Kao, 1970). In the future, exotic traits from an agronomical perspective such as a perennial growth habit or self‐fertility could be stably introduced into rye from the wild genepool. If incomplete lineage sorting also extends to structural variants such as chromosomal inversions, we can expect to find chromosomal rearrangements to underlie both apparently interspecies fertility barriers and occasional intraspecific hybrid sterility as has been observed in rye (Stutz, 1976). The construction of interspecific linkage maps and the application of novel methods for physical genome mapping such as chromosome conformation capture sequencing (Harewood et al., 2017) or optical mapping (Lam et al., 2012) are promising avenues for unraveling the causes for partial reproductive isolation between rye taxa, which may also shed light on the genetic basis of differences in the key characters self‐compatibility and annual life cycle.

In contrast to the situation in barley (Russell et al., 2016), we did not find a good correspondence between geography and genetic distance in rye. The predominant drivers of genetic differentiation in domesticated rye cannot be traced back to simple geographic gradients. *Bona fide* wild accessions cluster closely together in the PCA plot (Figure 4) and share ancestry proportions in an ADMIXTURE analysis (Figure 5). Parat et al. (2016) proposed multiple domestication origins and no apparent reduction in diversity in the crop based on microsatellite of domesticated and weedy ex situ accessions. Part of this complex relationship between wild and domesticated germplasm may be explained by (i) the purported late origin of rye as secondary domesticate from a noxious weed in wheat and barley in Europe (Behre, 1992) and (ii) its outcrossing breeding system. These two factors may have favored the capture of more ancestral genetic diversity from the wild progenitor into the domesticated genepools and facilitated crop‐wild gene flow. It is not unlikely that gene flow has been bidirectional, resulting in the genetic assimilation of wild‐growing and cultivated populations, which obfuscates the difference between “truly” wild, weedy, feral, and “fully” domesticated plants. Our small panel with only five accessions may not capture the full extent of population structure in *S. cereale* subsp. *vavilovii*. For instance, there may be many more “outlier” accessions such as R1003, which may actually represent rare unadmixed wild populations that would be highly informative on the domestication origin(s) of rye.

To summarize, our data are consistent with the notion that the domestication history of rye is more complex than that of the Neolithic founder crops wheat and barley. We believe that a more comprehensive assessment of the rye genetic diversity maintained in ex situ collections and, subsequently, a targeted effort toward broader sampling of wild and weedy populations are necessary to better understand the population structure in domesticated rye and its wild relatives.

## DATA ARCHIVING STATEMENT

Data for this study are available at the European Nucleotide Archive under accession number PRJEB22681 at the Plant Genomics and Phenomics Research Data Repository under the Digital Object Identifier https://doi.org/10.5447/ipk/2018/1.

## **CONFLICT OF INTEREST**

None declared.

## References

[eva12624-bib-0001] Abbo, S. , Lev‐Yadun, S. , Heun, M. , & Gopher, A. (2013). On the ‘lost’ crops of the neolithic Near East. Journal of Experimental Botany, 64, 815–822. 10.1093/jxb/ers373 23440172 PMC3594941

[eva12624-bib-0002] Alexander, D. H. , Novembre, J. , & Lange, K. (2009). Fast model‐based estimation of ancestry in unrelated individuals. Genome Research, 19, 1655–1664. 10.1101/gr.094052.109 19648217 PMC2752134

[eva12624-bib-0003] Arend, D. , Junker, A. , Scholz, U. , Schüler, D. , Wylie, J. , & Lange, M. (2016) PGP repository: a plant phenomics and genomics data publication infrastructure. Database 2016: baw033 10.1093/database/baw033 PMC4834206 27087305

[eva12624-bib-0004] Arend, D. , Lange, M. , Chen, J. , Colmsee, C. , Flemming, S. , Hecht, D. , & Scholz, U. (2014). e!DAL–a framework to store, share and publish research data. BMC Bioinformatics, 15, 214 10.1186/1471-2105-15-214 24958009 PMC4080583

[eva12624-bib-0005] Arnold, B. , Corbett‐Detig, R. B. , Hartl, D. , & Bomblies, K. (2013). RADseq underestimates diversity and introduces genealogical biases due to nonrandom haplotype sampling. Molecular Ecology, 22, 3179–3190. 10.1111/mec.12276 23551379

[eva12624-bib-0006] Bauer, E. , Schmutzer, T. , Barilar, I. , Mascher, M. , Gundlach, H. , Martis, M. M. , … Scholz, U. (2017). Towards a whole‐genome sequence for rye (*Secale cereale* L.). The Plant Journal, 89, 853–869. 10.1111/tpj.13436 27888547

[eva12624-bib-0007] Behre, K.‐E. (1992). The history of rye cultivation in Europe. Vegetation History and Archaeobotany, 1, 141–156.

[eva12624-bib-0008] Bhatia, G. , Patterson, N. , Sankararaman, S. , & Price, A. L. (2013). Estimating and interpreting FST: The impact of rare variants. Genome Research, 23, 1514–1521. 10.1101/gr.154831.113 23861382 PMC3759727

[eva12624-bib-0009] Camacho, C. , Coulouris, G. , Avagyan, V. , Ma, N. , Papadopoulos, J. , Bealer, K. , & Madden, T. L. (2009). BLAST+: Architecture and applications. BMC Bioinformatics, 10, 421 10.1186/1471-2105-10-421 20003500 PMC2803857

[eva12624-bib-0010] Chapman, J. A. , Mascher, M. , Buluc, A. N. , Barry, K. , Georganas, E. , Session, A. , … Rokhsar, D. S. (2015). A whole‐genome shotgun approach for assembling and anchoring the hexaploid bread wheat genome. Genome Biology, 16, 26 10.1186/s13059-015-0582-8 25637298 PMC4373400

[eva12624-bib-0011] Chikmawati, T. , Skovmand, B. , & Gustafson, J. P. (2005). Phylogenetic relationships among *Secale* species revealed by amplified fragment length polymorphisms. Genome, 48, 792–801. 10.1139/g05-043 16391685

[eva12624-bib-0012] Devos, K. , Millan, T. , & Gale, M. (1993). Comparative RFLP maps of the homoeologous group‐2 chromosomes of wheat, rye and barley. TAG Theoretical and Applied Genetics, 85, 784–792. 24196051 10.1007/BF00225020

[eva12624-bib-0013] Elshire, R. J. , Glaubitz, J. C. , Sun, Q. , Poland, J. A. , Kawamoto, K. , Buckler, E. S. , & Mitchell, S. E. (2011). A robust, simple genotyping‐by‐sequencing (GBS) approach for high diversity species. PLoS ONE, 6, e19379 10.1371/journal.pone.0019379 21573248 PMC3087801

[eva12624-bib-0014] Frederiksen, S. , & Petersen, G. (1998). A taxonomic revision of *Secale* (Triticeae, Poaceae). Nordic Journal of Botany, 18, 399–420. 10.1111/j.1756-1051.1998.tb01517.x

[eva12624-bib-0015] Gautier, M. , Gharbi, K. , Cezard, T. , Foucaud, J. , Kerdelhue, C. , Pudlo, P. , … Estoup, A. (2013). The effect of RAD allele dropout on the estimation of genetic variation within and between populations. Molecular Ecology, 22, 3165–3178. 10.1111/mec.12089 23110526

[eva12624-bib-0016] Hagenblad, J. , Oliveira, H. R. , Forsberg, N. E. G. , & Leino, M. W. (2016). Geographical distribution of genetic diversity in *Secale landrace* and wild accessions. BMC Plant Biology, 16, 23 10.1186/s12870-016-0710-y 26786820 PMC4719562

[eva12624-bib-0017] Hammer, K. (1990). Breeding system and phylogenetic relationships in *Secale* L. Biologisches Zentralblatt, 109, 45–50.

[eva12624-bib-0018] Hammer, K. , Skolimowska, E. , & Knüpffer, H. (1987). Vorarbeiten zur monographischen Darstellung von Wildpflanzensortimenten: *Secale* L. Die Kulturpflanze, 35, 135–177. 10.1007/BF02113274

[eva12624-bib-0019] Harewood, L. , Kishore, K. , Eldridge, M. D. , Wingett, S. , Pearson, D. , Schoenfelder, S. , … Fraser, P. (2017). Hi‐C as a tool for precise detection and characterisation of chromosomal rearrangements and copy number variation in human tumours. Genome Biology, 18, 125 10.1186/s13059-017-1253-8 28655341 PMC5488307

[eva12624-bib-0020] Harlan, J. R. , & de Wet, J. M. J. (1971). Toward a rational classification of cultivated plants. Taxon, 20, 509–517. 10.2307/1218252

[eva12624-bib-0021] Harris, K. , & Nielsen, R. (2013). Inferring demographic history from a spectrum of shared haplotype lengths. PLOS Genetics, 9, e1003521 10.1371/journal.pgen.1003521 23754952 PMC3675002

[eva12624-bib-0022] Hartyányi, B. P. , & Novaki, G. (1975), Samen‐ und Fruchtfunde in Ungarn von der Neusteinzeit bis zum 18. Jahrhundert. Agrartorteneti Szemle, 17(suppl.), 1–88. (in German).

[eva12624-bib-0023] Haseneyer, G. , Schmutzer, T. , Seidel, M. , Zhou, R. , Mascher, M. , Schon, C. C. , … Bauer, E. (2011). From RNA‐seq to large‐scale genotyping ‐ genomics resources for rye (*Secale cereale* L.). BMC Plant Biology, 11, 131 10.1186/1471-2229-11-131 21951788 PMC3191334

[eva12624-bib-0024] Hillman, G. (1978). On the origins of domestic rye—*Secale cereale*: The finds from aceramic Can Hasan III in Turkey. Anatolian Studies, 28, 157–174. 10.2307/3642748

[eva12624-bib-0025] Houben, A. , Sanei, M. , & Pickering, R. (2011). Barley doubled‐haploid production by uniparental chromosome elimination. Plant Cell Tissue and Organ Culture (PCTOC): Journal of Plant Biotechnology, 104, 321–327. 10.1007/s11240-010-9856-8

[eva12624-bib-0026] Hrishi, N. J. , & Müntzing, A. (1960). Structural heterozygosity IN *Secale kuprijanovii* . Hereditas, 46, 745–752.

[eva12624-bib-0027] Hudson, R. R. , Slatkin, M. , & Maddison, W. P. (1992). Estimation of levels of gene flow from DNA sequence data. Genetics, 132, 583–589. 1427045 10.1093/genetics/132.2.583 PMC1205159

[eva12624-bib-0028] International Barley Genome Sequencing Consortium (2012). A physical, genetic and functional sequence assembly of the barley genome. Nature, 491, 711–716. 23075845 10.1038/nature11543

[eva12624-bib-0029] International Wheat Genome Sequencing Consortium (2014). A chromosome‐based draft sequence of the hexaploid bread wheat (*Triticum aestivum*) genome. Science, 345, 1251788. 25035500 10.1126/science.1251788

[eva12624-bib-0030] Jakob, S. S. , Rodder, D. , Engler, J. O. , Shaaf, S. , Ozkan, H. , Blattner, F. R. , & Kilian, B. (2014). Evolutionary history of wild barley (*Hordeum vulgare* subsp. *spontaneum*) analyzed using multilocus sequence data and paleodistribution modeling. Genome Biology and Evolution, 6, 685–702. 10.1093/gbe/evu047 24586028 PMC3971598

[eva12624-bib-0031] Jakobsson, M. , & Rosenberg, N. A. (2007). CLUMPP: A cluster matching and permutation program for dealing with label switching and multimodality in analysis of population structure. Bioinformatics, 23, 1801–1806. 10.1093/bioinformatics/btm233 17485429

[eva12624-bib-0032] Jombart, T. , & Ahmed, I. (2011). adegenet 1.3‐1: New tools for the analysis of genome‐wide SNP data. Bioinformatics, 27, 3070–3071. 10.1093/bioinformatics/btr521 21926124 PMC3198581

[eva12624-bib-0033] Jordan, K. W. , Wang, S. , Lun, Y. , Gardiner, L. J. , MacLachlan, R. , Hucl, P. , … Akhunov, E. (2015) A haplotype map of allohexaploid wheat reveals distinct patterns of selection on homoeologous genomes. Genome Biology 16: 48 10.1186/s13059-015-0606-4 25886949 PMC4389885

[eva12624-bib-0034] Kasha, K. , & Kao, K. (1970). High frequency haploid production in barley (*Hordeum vulgare* L.). Nature, 225, 874–876. 10.1038/225874a0 16056782

[eva12624-bib-0035] Khush, G. S. (1962). Cytogenetic and evolutionary studies in *Secale*. II Interrelationships of the wild species. Evolution, 16, 484–496.

[eva12624-bib-0036] Khush, G. S. , & Stebbins, G. L. (1961). Cytogenetic and evolutionary studies in *Secale*. I. Some new data on the ancestry of *S. cereale* . American Journal of Botany, 48, 723–730. 10.1002/j.1537-2197.1961.tb11703.x

[eva12624-bib-0037] Lam, E. T. , Hastie, A. , Lin, C. , Ehrlich, D. , Das, S. K. , Austin, M. D. , … Kwok, P. Y. (2012). Genome mapping on nanochannel arrays for structural variation analysis and sequence assembly. Nature Biotechnology, 30, 771–776. 10.1038/nbt.2303 PMC3817024 22797562

[eva12624-bib-0038] Li, H. (2011). A statistical framework for SNP calling, mutation discovery, association mapping and population genetical parameter estimation from sequencing data. Bioinformatics, 27, 2987–2993. 10.1093/bioinformatics/btr509 21903627 PMC3198575

[eva12624-bib-0039] Li, H. . (2013) Aligning sequence reads, clone sequences and assembly contigs with BWA‐MEM. arXiv preprint arXiv:1303.3997

[eva12624-bib-0040] Li, H. , Handsaker, B. , Wysoker, A. , Fennell, T. , Ruan, J. , Homer, N. , … Genome Project Data Processing S. (2009) The Sequence Alignment/Map format and SAMtools. Bioinformatics, 25, 2078–2079. 10.1093/bioinformatics/btp352 19505943 PMC2723002

[eva12624-bib-0041] Li, H. , Vikram, P. , Singh, R. P. , Kilian, A. , Carling, J. , Song, J. , … Singh, S. (2015). A high density GBS map of bread wheat and its application for dissecting complex disease resistance traits. BMC Genomics, 16, 216 10.1186/s12864-015-1424-5 25887001 PMC4381402

[eva12624-bib-0042] Martin, M. (2011). Cutadapt removes adapter sequences from high‐throughput sequencing reads. EMBnet. Journal, 17, 10–12. 10.14806/ej.17.1.200

[eva12624-bib-0043] Mascher, M. , Wu, S. , Amand, P. S. , Stein, N. , & Poland, J. (2013). Application of genotyping‐by‐sequencing on semiconductor sequencing platforms: A comparison of genetic and reference‐based marker ordering in barley. PLoS ONE, 8, e76925 10.1371/journal.pone.0076925 24098570 PMC3789676

[eva12624-bib-0044] McElroy, J. S. (2014). Vavilovian mimicry: Nikolai Vavilov and his little‐known impact on weed science. Weed science, 62, 207–216. 10.1614/WS-D-13-00122.1

[eva12624-bib-0045] Milczarski, P. , Hanek, M. , Tyrka, M. , & Stojałowski, S. (2016). The application of GBS markers for extending the dense genetic map of rye (*Secale cereale* L.) and the localization of the Rfc1 gene restoring male fertility in plants with the C source of sterility‐inducing cytoplasm. Journal of Applied Genetics, 57, 439–451. 10.1007/s13353-016-0347-4 27085345 PMC5061839

[eva12624-bib-0046] Nesbitt, M. (2002) When and where did domesticated cereals first occur in southwest Asia. The dawn of farming in the Near East: 113‐132.

[eva12624-bib-0047] Oppermann, M. , Weise, S. , Dittmann, C. , & Knüpffer, H. (2015). GBIS: The information system of the German Genebank. Database, 2015, bav021, 10.1093/database/bav021 PMC4423411 25953079

[eva12624-bib-0048] Paradis, E. , Claude, J. , & Strimmer, K. (2004). APE: Analyses of phylogenetics and evolution in R language. Bioinformatics, 20, 289–290. 10.1093/bioinformatics/btg412 14734327

[eva12624-bib-0049] Parat, F. , Schwertfirm, G. , Rudolph, U. , Miedaner, T. , Korzun, V. , Bauer, E. , … Tellier, A. (2016). Geography and end use drive the diversification of worldwide winter rye populations. Molecular ecology, 25, 500–514. 10.1111/mec.13495 26607414

[eva12624-bib-0050] Patterson, N. , Moorjani, P. , Luo, Y. , Mallick, S. , Rohland, N. , Zhan, Y. , … Reich, D. (2012). Ancient admixture in human history. Genetics, 192, 1065–1093. 10.1534/genetics.112.145037 22960212 PMC3522152

[eva12624-bib-0051] Patterson, N. , Price, A. L. , & Reich, D. (2006). Population structure and eigenanalysis. PLoS Genetics, 2, e190 10.1371/journal.pgen.0020190 17194218 PMC1713260

[eva12624-bib-0052] Perrino, P. , Hammer, K. , & Hanelt, P. (1984). Collection of land‐races of cultivated plants in South Italy 1983. Die Kulturpflanze, 32, 207–216. 10.1007/BF02002078

[eva12624-bib-0053] Petersen, G. , & Doebley, J. F. (1993). Chloroplast DNA variation in the genus *Secale* (Poaceae). Plant Systematics and Evolution, 187, 115–125. 10.1007/BF00994094

[eva12624-bib-0054] Poland, J. A. , Brown, P. J. , Sorrells, M. E. , & Jannink, J.‐L. (2012). Development of high‐density genetic maps for barley and wheat using a novel two‐enzyme genotyping‐by‐sequencing approach. PLoS ONE, 7, e32253 10.1371/journal.pone.0032253 22389690 PMC3289635

[eva12624-bib-0055] Preece, C. , Livarda, A. , Christin, P.‐A. , Wallace, M. , Martin, G. , Charles, M. , … Osborne, C. P. (2017). How did the domestication of fertile crescent grain crops increase their yields? Functional Ecology, 31, 387–397. 10.1111/1365-2435.12760 28286354 PMC5324541

[eva12624-bib-0056] Quinlan, A. R. , & Hall, I. M. (2010). BEDTools: A flexible suite of utilities for comparing genomic features. Bioinformatics, 26, 841–842. 10.1093/bioinformatics/btq033 20110278 PMC2832824

[eva12624-bib-0057] R Core Team (2015) R: A Language and Environment for Statistical Computing. Vienna, Austria: R Foundation for Statistical Computing.

[eva12624-bib-0058] Rakoczy‐Trojanowska, M. , Krajewski, P. , Bocianowski, J. , Schollenberger, M. , Wakuliński, W. , Milczarski, P. , … Kilian, A. (2017). Identification of single nucleotide polymorphisms associated with brown rust resistance, α‐amylase activity and pre‐harvest sprouting in rye (*Secale cereale* L.). Plant Molecular Biology Reporter, 35, 366–378. 10.1007/s11105-017-1030-6 28603340 PMC5443880

[eva12624-bib-0059] Reich, D. , Thangaraj, K. , Patterson, N. , Price, A. L. , & Singh, L. (2009). Reconstructing Indian population history. Nature, 461, 489–494. 10.1038/nature08365 19779445 PMC2842210

[eva12624-bib-0060] Roshevitz, R. (1947). A monograph of the wild, weedy and cultivated species of rye. Acta Inst Bot Nomine Acad Sci USSR Ser 1, 105–163.

[eva12624-bib-0061] Russell, J. , Mascher, M. , Dawson, I. K. , Kyriakidis, S. , Calixto, C. , Freund, F. , Bayer, M. , Milne, I. , Marshall‐Griffiths, T. , Heinen, S. , & Waugh, R. (2016). Exome sequencing of geographically diverse barley landraces and wild relatives gives insights into environmental adaptation. Nature Genetics, 48(9), 1024–1030. 10.1038/ng.3612 27428750

[eva12624-bib-0062] Singh, R. J. (1977). Cross compatibility, meiotic pairing and fertility in 5 *Secale* species and their interspecific hybrids. Cereal Research Communications, 5, 67–75.

[eva12624-bib-0063] Singh, R. , & Röbbelen, G. (1977). Identification by Giemsa technique of the translocations separating cultivated rye from three wild species of *Secale* . Chromosoma, 59, 217–225. 10.1007/BF00292779

[eva12624-bib-0064] Stutz, H. C. (1957). A cytogenetic analysis of the hybrid secale cereale l. x secale montanum guss. and its progeny. Genetics, 42, 199. 17247691 10.1093/genetics/42.3.199 PMC1209825

[eva12624-bib-0065] Stutz, H. C. (1976). Genetically controlled chromosome breakage as an isolation barrier in the origin and maintenance of Secale ancestrale. Canadian Journal of Genetics and Cytology, 18, 105–109. 10.1139/g76-015

[eva12624-bib-0066] Voylokov, A. V. , Fuong, F. T. , & Smirnov, V. G. (1993). Genetic studies of self‐fertility in rye (Secale cereale L.). 1. The identification of genotypes of self‐fertile lines for the Sf alleles of self‐incompatibility genes. TAG. Theoretical and Applied Genetics., 87, 616–618. 24190358 10.1007/BF00221887

[eva12624-bib-0067] Wendler, N. , Mascher, M. , Himmelbach, A. , Johnston, P. , Pickering, R. , & Stein, N. (2015). Bulbosum to go: A toolbox to utilize *Hordeum vulgare/bulbosum* Introgressions for Breeding and Beyond. Molecular Plant, 8, 1507–1519. 10.1016/j.molp.2015.05.004 25983208

[eva12624-bib-0068] Wendler, N. , Mascher, M. , Noh, C. , Himmelbach, A. , Scholz, U. , Ruge‐Wehling, B. , & Stein, N. (2014). Unlocking the secondary gene‐pool of barley with next‐generation sequencing. Plant Biotechnology Journal, 12, 1122–1131. 10.1111/pbi.12219 25040223

[eva12624-bib-0069] Zheng, X. , Gogarten, S. M. , Lawrence, M. , Stilp, A. , Conomos, M. P. , Weir, B. S. , … Levine, D. (2017). SeqArray‐a storage‐efficient high‐performance data format for WGS variant calls. Bioinformatics, 33, 2251–2257. 10.1093/bioinformatics/btx145 28334390 PMC5860110

[eva12624-bib-0070] Zheng, X. , Levine, D. , Shen, J. , Gogarten, S. M. , Laurie, C. , & Weir, B. S. (2012). A high‐performance computing toolset for relatedness and principal component analysis of SNP data. Bioinformatics, 28, 3326–3328. 10.1093/bioinformatics/bts606 23060615 PMC3519454

[eva12624-bib-0071] Zohary, D. (1960) Spontaneous brittle six‐row barleys, their nature and origin.

[eva12624-bib-0072] Zohary, D. , Hopf, M. , & Weiss, E. (2012) Domestication of Plants in the Old World: The origin and spread of domesticated plants in Southwest Asia, Europe, and the Mediterranean Basin. Oxford University Press on Demand

