# Supplementary material for: Genetic diversity and relationship between domesticated rye and its wild relatives as revealed through genotyping‐by‐sequencing
Source: Evol Appl. 2018 Mar 26;12(1):66–77. doi: 10.1111/eva.12624 (PMC6304746; doi:10.1111/eva.12624)
Supplement: Supplementary file 1 [file EVA-12-66-s001.pptx]

## Slide 1
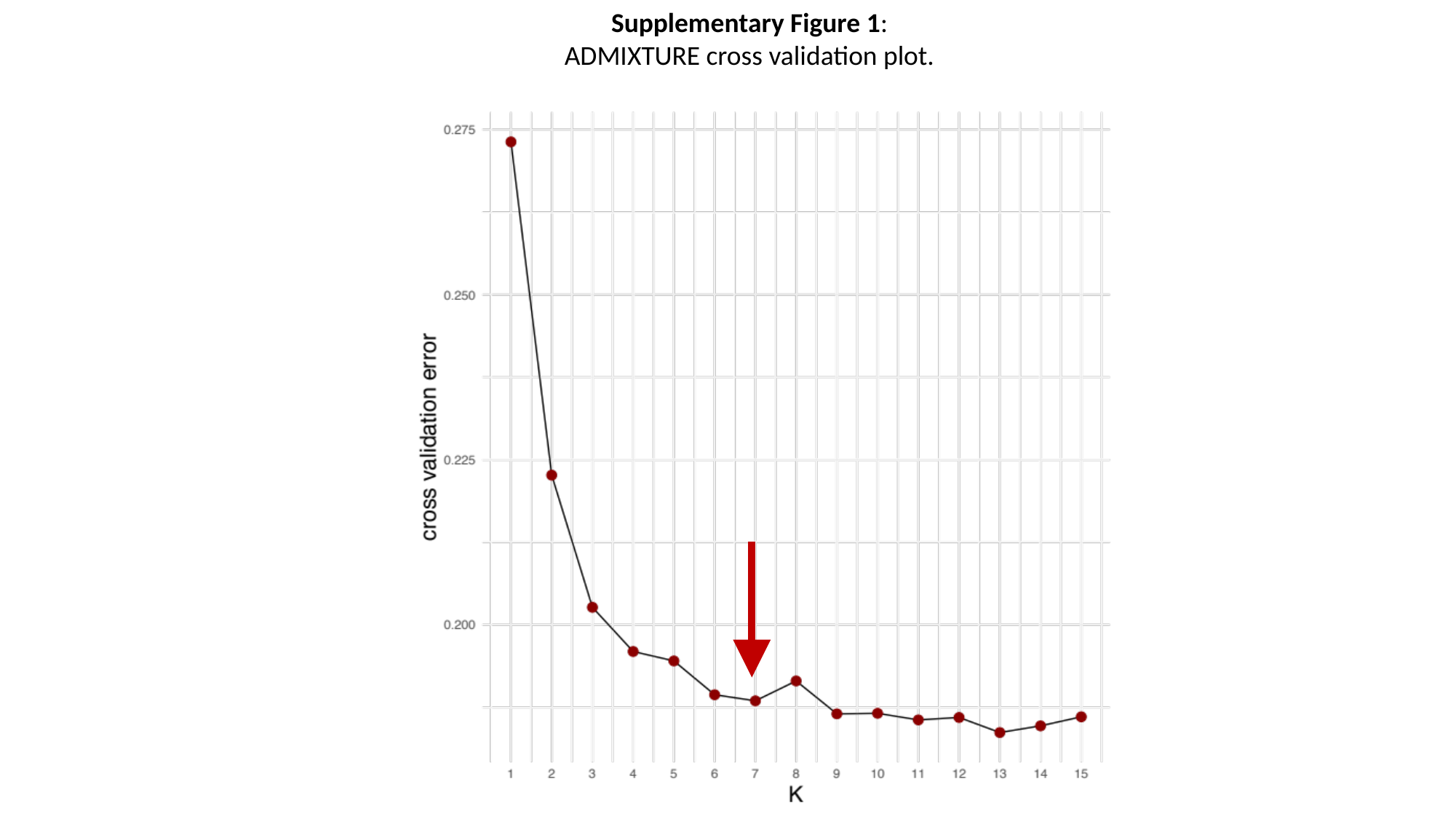

Supplementary Figure 1:
ADMIXTURE cross validation plot.

## Slide 2
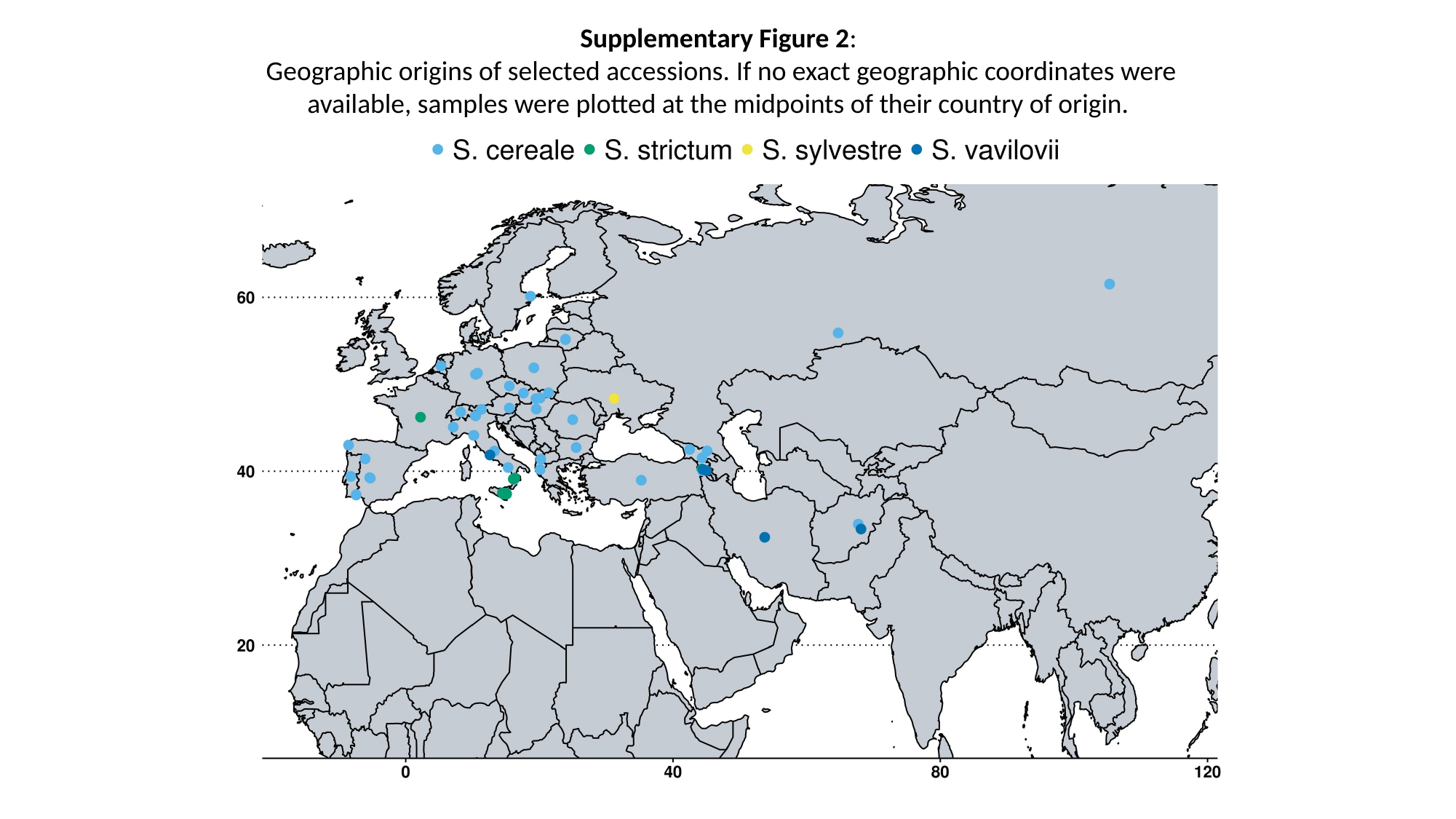

Supplementary Figure 2:
Geographic origins of selected accessions. If no exact geographic coordinates were available, samples were plotted at the midpoints of their country of origin.
